# Dates fruit effects on dyslipidemia among patients with Type-2 diabetes mellitus: A systematic review and meta-analysis of randomized trials

**DOI:** 10.12669/pjms.41.1.9928

**Published:** 2025-01

**Authors:** Hyder Osman Mirghani, Amirah Alhowiti

**Affiliations:** 1 Hyder Osman Mirghani Professor of Internal Medicine and Endocrine, Department of Internal Medicine, Faculty of Medicine, University of Tabuk, Saudi Arabia; 2 Amirah Alhowiti Assistant Professor of Family Medicine, Department of Family and Community Medicine, Faculty of Medicine, University of Tabuk, Saudi Arabia

**Keywords:** Dates fruit, Phoenix dactylifera, Dyslipidemia, Diabetes

## Abstract

**Objectives:**

Dyslipidemias are major risk factors for cardiovascular disease, and other comorbidities. The focus on food and nutrition to prevent and treat cardiovascular risk factors including dyslipidemia is a paradigm shift. This is the first meta-analysis to assess the association of dates fruit and dyslipidemia in Type-2 diabetes. The study aimed to assess the same among patients with Type-2 diabetes.

**Methods:**

Six databases were searched for relevant articles from inception to March 2024. We included randomized trials, studies with other methods, and those conducted among healthy people were excluded. A structured checklist including the author’s name, country, year of publication, study type, duration of the study, lipid profile at baseline and after-dates fruit consumption, age, and gender of participants, type of dates, and the amount consumed.

**Results:**

Out of the 448 studies retrieved, fourteen cohorts from four studies (298 participants with Type-2 diabetes) were included. Dates fruit reduced cholesterol, odd ratio, -0.87, 95% *CI*, -1.39--0.35, P-value, 0.001, and *I^2^* for heterogeneity=90%. No significant changes were observed on low-density lipoprotein (odd ratio, -0.31, 95% *CI*, -0.65-0.03, and P-value, 0.08), triglycerides (odd ratio, -0.77, 95% *CI*, -2.17--0.63, P-value 0.28), and high-density lipoproteins (odd ratio, 0.03, 95% *CI*, -0.13-0.19, P-value, 0.69). The *I^2^* for heterogeneity were=99%, 95%, and 65% respectively.

**Conclusion:**

Dates fruit could reduce total cholesterol, with a non-significant reduction in low-density lipoproteins. No significant effect was evident regarding triglycerides and high-density lipoproteins. Further larger studies with a high selection of controls and dates are needed.

## INTRODUCTION

Dyslipidemia is major risk factor for cardiovascular disease and target organ damage including the pancreas and the liver. Therefore, low-density lipoprotein (LDL) was the fifteenth cause of death in the nineties of the previous century and jumps to the eighth by the year 2019.^1^ Dyslipidemia is a major risk factor for cardiovascular disease; the disease is on the rise globally in particular during the previous three decades. As a major component of metabolic syndrome, dyslipidemias are major determinants of morbidity and cardiovascular mortality. Although, cholesterol metabolism is internal (by the liver), but atherogenic dyslipidemia (dense particle low-density lipoproteins, high triglycerides, and low high-density lipoproteins) are significantly affected by lifestyles.^2^,^3^ Diabetes mellitus is reaching an epidemic and the prevalence is rising worldwide. In addition, the cross talk between diabetes mellitus and dyslipidemia was documented, when these two major cardiovascular risk factors co-exist, they facilitate each other deleterious consequences.^4^ Therefore, treating dyslipidemia among patients with diabetes is highly important to reduce cardiovascular disease.^5^

Healthy lifestyles including the adoption of friendly diet are strongly recommended for patients with diabetes and dyslipidemia.^6^ Fruits are important source of energy, fiber, potassium, and vitamins. In addition, evidence from observational studies showed that high fruit consumption is associated with lower rates of diabetes and other cardiometabolic risk factors. Fruits, legumes, and vegetable consumption reduce both total and non-cardiovascular mortality,^7^ because of this, many guidelines recommend the consumption of 400-800 grams of fruits and vegetables daily.

Dates fruit (Phoenix dactylifera) is widely available and cheap food. Because of its high nutritional value, two/three servings per day of date’s fruit are beneficial for healthy people and those suffering from diabetes. Dates fruit consumption is associated with good glycemic control among patients with diabetes and healthy people without diabetes. In addition, High glucose load can alter the lipid generation in the body and affect blood lipid levels. Therefore, dates fruit consumption should be restricted to the daily recommended fruits servings^8^,^9^ Animal studied reported the positive influence of dates fruit on dyslipidemias.^10^ Plausible explanations are the influence of dates fruit on inflammation and oxidative stress biomarkers.^11^ Literature evaluating the effects of date’s fruit on dyslipidemia among patients with diabetes mellitus is scarce.^12^ To the best of our knowledge, no meta-analysis has assessed the effects of date fruit on dyslipidemia. Thus, we conducted this meta-analysis to evaluate the impact of date fruit on dyslipidemia among patients with Type-2 diabetes mellitus.

## METHODS

This meta-analysis was conducted during February and March 2024. The authors strictly followed the PRISMA Guidelines. We include randomized trials because of the high level of evidence. Other observational studies, and animal studies, case series, editorials, experts’ opinions were excluded due to the low-level of evidence.

### Inclusion criteria:

- Studies were included if they were randomized trials assessing the effects of dates fruit on lipid profile among patients with Type-2 diabetes.

### Exclusion criteria:

- Retrospective studies, prospective cohorts, case-control, and cross-sectional studies were not included. In addition, case series, editorials, experts’ opinions, and reviews were excluded. Studies investigating the effects of date fruit on dyslipidemia among healthy subjects and Type-1 diabetes were excluded due to the different metabolism.

### Outcome measures:

The outcome measures were the effects of date’s fruit on total cholesterol, triglycerides, low-density lipoproteins, and high-density lipoproteins among patients with Type-2 diabetes.

### Literature search:

Two authors (H. M. and A. A.) independently searched six databases (Web of Science, SCOPUS, PubMed, MEDLINE, Google Scholar, Cochrane Library, and EBSCO). The literature search was conducted during February and March 2024. The search engine was set for articles published in English from inception to the recently published article. The keywords used were cholesterol, low-density lipoproteins, high-density lipoproteins, triglycerides, lipid profile, dates fruit, and Phoenix dactylifera. Four hundred and forty-eight studies were retrieved, and one hundred and sixty-seven remained after duplication removal, of them 27 full texts were screened. However, only four studies fulfilled the inclusion and exclusion criteria. During literature search and data extraction, any discrepancies were solved by an agreement.

### Data extraction:

**Fig.1:**
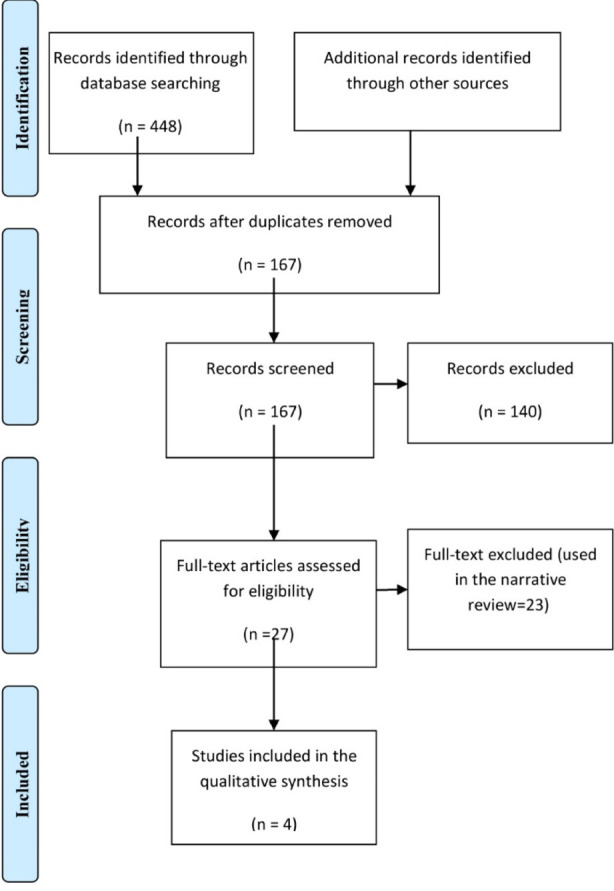
Randomized trials were included in the meta-analysis (The PRISMA Chart).

A structured checklist was used to gather the author’s name, country, year of publication, study type, duration of the study, lipid profile at baseline and after-dates fruit consumption, and type of dates and the amount consumed. Importantly, the different types of dates fruits consumed had different glycemic index. The different content of dates fruit used could add to the variability of this results. The checklist also included the age, sex, type and number of dates consumed and the study duration. Fig.1 & Table-I-III.

**Table-I:** Characteristics of dates and patients with diabetes.

Author	Age/years	Females%	Dates type and amount of dates	Number of patients	Study duration
Al-Abachi et al. 2022^14^	47.7±9.33	50%	Zahdi dates (5-7) before breakfasting	Twenty	Three weeks
Alalwan et al. 2020^15^	55.25 ± 2.71	61%	Khudary (local, Bahrain), 3 dates/day	Fifty	Sixteen weeks
Ali 2018 et al.^16^	46.4 ± 1.5	53.6%	Dates vinegar, 20 ml/day	Fifty-five	10 weeks
Butler 2022 et al.^17^	61	50.6%,	60 grams dates	Twenty	Four weeks

**Table-II:** Effects of dates fruit on cholesterol and low-density lipoproteins (LDL) among patients with diabetes.

Author	Country	Cholesterol before dates consumption	Cholesterol after dates consumption	LDL before dates consumption	LDL after dates consumption
Al-Abachi et al. 2022^14^	Iraq	6.94±0.84	5.32±0.41	Not assessed	Not assessed
Alalwan et al. 2020^15^	Bahrain	4.127 ± 1.04	3.918 ± 1.04	2.3035 ± 0.92	2.1325 ± 0.92
Ali 2018 et al.^16^	Pakistan	5.64 ± 0.1	4.94 ± 0.1	3.04±0.1	2.52±0.12
Butler 2022 et al.^17^	Bahrain	4.9±1.4	3.9±1.0	2.6±0.9	2.7±1.4

**Table-III:** Effects of dates fruit on triglycerides (TG), and high-density lipoproteins (HDL) among patients with diabetes.

Author	Country	TG before dates intake	TG after dates intake	HDL Before dates intake	HDL after dates intake
Al-Abachi et al. 2022^14^	Iraq	5.35±0.69	2.48±0.24	Not assessed	Not assessed
Alalwan et al. 2020^15^	Bahrain	1.693 ± 0.88	1.689 ± 0.88	1.149 ± 0.30	1.072 ± 0.30
Ali 2018 et al.^16^	Pakistan	2.41±0.05	2.34±0.05	1.08±0.12	1.23±0.12
Butler 2022 et al.^17^	Bahrain	1.6±1.2	1.5±0.7	1.1±0.2	1.1±0.3

### Risk of bias assessment:

The Cochrane risk of bias assessment tool (Rob-2) was used.^13^ Table-IV.

**Table-IV:** Risk of bias (Rob-2) assessment of the included studies according to Cochrane risk of bias of randomized controlled trials.

Author	Selection bias^1^	Selection bias^2^	Performance bias	Attrition bias	Detection bias	Reporting bias	Overall bias
Al-Abachi et al. 2022^14^	Some concern	Some concern	High	Low	High	Low	Some concern
Alalwan et al. 2020^15^	Low	Low	Some concerns	Low	Some concerns	Low	Low
Ali 2018 et al.^16^	Low	Low	Some concerns	Low	Some concerns	Low	Low
Butler 2022 et al.^17^	Low	Low	Some concerns	Low	Some concerns	Low	Low

### Statistical analysis:

The last version of RevMan system for meta-analysis, version, 5.4 was used to analyze the continuous data assessing the effect of date’s fruit on cholesterol, low-density lipoproteins, high-density lipoproteins, and triglycerides. The mean differences and 95% confidence intervals were applied. The random effect was used because of the significant heterogeneity observed, The *I^2^* for heterogeneity were 90% for cholesterol, 99% for low-density lipoproteins, 95% for triglycerides, and 65% for low-density lipoproteins. A sub-analysis was conducted to address the heterogeniety, and two references were eliminated with no change in the results except for the triglyceride arm in which dates fruit reduced triglycerides after eliminating the heterogeniety. A P-value of < 0.05 was considered significant.

## RESULTS

### Characteristics of the included studies:

All the studies were published in Asia (50% to 61% were women), their age ranged from 46.4 ± 1.5 to 61 years, and the number of dates varied between three dates to 60 grams and one of the studies used dates vinegar. The duration of the included studies ranged from 3-16 weeks and the number of participants was 298, Table-I.

### Findings:

**Fig.2:**
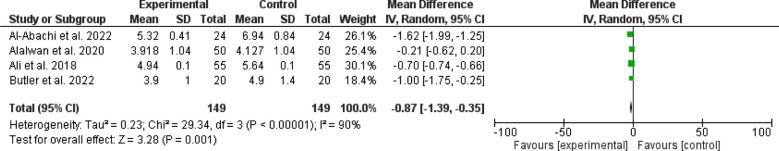
Effects of dates fruit on total cholesterol among patients with diabetes.

In the present meta-analysis, we included fourteen cohorts from four studies^14^-^17^ (298 patients with Type-2 diabetes). All the studies were randomized controlled trials and published in Asia. Dates fruit reduced cholesterol, odd ratio, -0.87, 95% *CI*, -1.39--0.35, P-value, 0.001, and Chi-square=29.34. Significant heterogeneity was found, *I^2^*for heterogeneity=90%, P-value for heterogeneity <0.001, and the standard difference=three, Fig.2.

**Fig.3:**
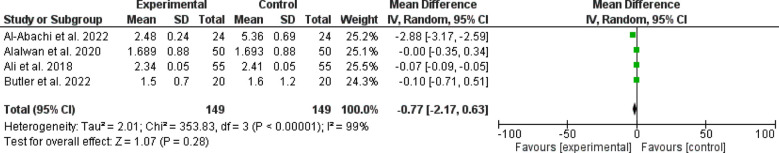
Effects of dates fruit on triglycerides among patients with diabetes.

**Fig.4:**
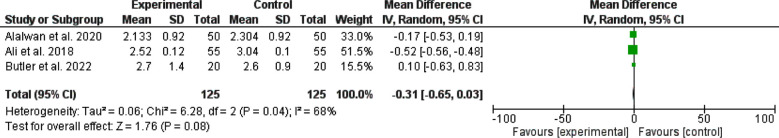
Effects of dates fruit on low-density lipoproteins among patients with diabetes.

**Fig.5:**
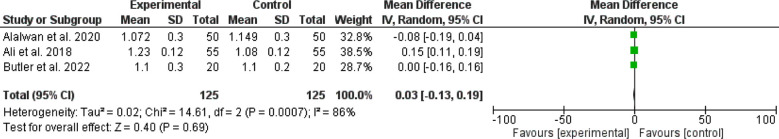
Effects of dates fruit on high-density lipoproteins among patients with diabetes.

Regarding triglyceride, no significant reduction was found, odd ratio, -0.77, 95% *CI*, -2.17--0.63, P-value 0.28, and Chi-square=353.83. Significant heterogeneity was found, *I^2^*for heterogeneity=99%, P-value for heterogeneity <0.001, and the standard difference=three. Fig.3. Dates fruit consumption showed no effects on low-density lipoprotein, odd ratio, -0.31, 95% *CI*, -0.65-0.03, P-value, 0.08, and Chi-square=6.28. Significant heterogeneity was found, *I^2^*for heterogeneity=68%, P-value for heterogeneity, 0.04, and the standard difference=two. Fig.4. No statistically significant change was found regarding high-density lipoproteins, odd ratio, 0.03, 95% *CI*, -0.13-0.19, P-value, 0.69, and Chi-square=14.61. Significant heterogeneity was found, *I^2^*for heterogeneity=86%, P-value for heterogeneity, 0.0007, and the standard difference=two, Fig.5.

## DISCUSSION

Dates fruit is very rich in trace elements, polyphenols, and phytochemicals, the fruit and due to its antioxidant capacity and effects on tumor necrosis factor expression; had the potential to mitigate DNA damage.^18^ Therefore, dates fruit is suitable for patients with diabetes due to the high prevalence of malnutrition observed among them.^19^ In addition, animal studies showed that date extracts are beneficial in dyslipidemia (lower low-density lipoproteins, total cholesterol, and triglycerides, and higher high-density lipoproteins).^20^,^12^

Furthermore, dates fruits inhibited LDL oxidation and removed cholesterol from macrophages with positive influence on cardiovascular disease.^21^ This meta-analysis showed that date fruits reduce total cholesterol among patients with diabetes with significant statistical differences. While the lowering potential on LDL was statistically significant. Previous studies among patients with diabetes showed the beneficial effects of date fruit consumption on quality of life and glycemic control.^10^,^20^ Data regarding the effects of dates fruit on dyslipidemia scarce. To date, we found only one recent narrative review that observed lowering effects of dates fruit on total cholesterol.^21^

The current findings included four randomized trials and supported the previous observation, which include only one trial. Dates fruits contain more than fifteen minerals including calcium, potassium, and zinc, in addition to 23 amino acids in contrast to many popular fruits including bananas, oranges, and apples.^22^ Dates fruit is a very rich source of saturated and unsaturated fatty acids, fiber, and vitamins.

### Benefits of dates fruit consumption:

Dates fruits are ideal food and have the potential to improve immunity, protect against cancer, and protect against cardiovascular risk factors including dyslipidemia, and hypertension.^23^ Importantly, poor glycemic control predicts dyslipidemia and microvascular complications. Therefore, dates fruit effects on lipids could be mediated in part by lowering blood glucose.^24^,^25^ We found a lowering effect of dates fruit on fasting and postprandial blood glucose in our previous meta-analysis on the effects of dates fruit on glycemic control.^8^ The wider benefits of date’s fruit among patients with diabetes include protection from cardiomyopathy,^26^ infections,^27^ diabetic neuropathy,^28^ and Alzheimer’s disease.^29^

### Dates fruit and the gut:

The effects of dates start from the gastrointestinal tract, and were shown to have a stimulating role in the GIT tract activity, and prevent constipation.^30^ An important issue is the effects on gut microbiota and stool constituents, dates fruit consumption effects on gut microbiota is contradicting with some studies showing no effect, while others observed a change in microbiota diversity (increased growth of bifidobacteria).^31^,^32^ Polyphenols and dietary fiber in date’s fruit are fermented by the gut microbiota increasing its diversity. Microbiota modulation is associated with lipid lowering in particular among patients with diabetes, dyslipidemia, and metabolic syndrome.^33^,^34^

### Polyphenols effects on lipids and cardiovascular health:

Proanthocyanidin isolated from dates fruit in California act as co-agonists of farnesoid x receptor which plays an important role in cholesterol and triglycerides homeostasis.^35^ In addition, polyphenols reduce scavenge radicals and ferric iron, inhibit LDL-C oxidation, and remove cholesterol from the macrophages.^36^

### Dietary fiber:

Most dates fiber is insoluble and bind to cholesterol in the gut and reduce its absorption exerting a lipid-lowering effects, animal studies observed lower cholesterol, low-density lipoproteins, and triglycerides in rats given 100 g/kg of date fibers.^37^ Fermentation of soluble fibers enhances short-chain fatty acids, and decreasing peroxisome proliferator-activated receptor expression shifts the metabolism in the liver and adipose tissue towards fatty acid oxidation.^38^,^39^

### Future research:

Data on the effects of dates on cardiovascular risk factors is lacking, unlike other fruits.^40^,^41^ Future studies on the same (selecting patients at risk) with proper identification of suitable controls and using control product that is closely matched to dates in term of taste, energy, and constituents are needed.

### Safety concerns:

Although many heavy metals in dates are within the recommended levels set by the World Health Organization. However, cadmium, lead, and arsenic levels exceeded the recommendations in some types of date fruit.^42^,^43^ Exposure to such heavy metals might increase cardiac disease, renal disease, dementia, and some cancers.^44^,^45^

### Clinical implications:

Because of the high heterogeneity observed in this meta-analysis and the lack of a significant overall effect. In addition, this meta-analysis included only patients with Type-2 diabetes and we did not assess the effects of dates fruit on glycemic control. The is no current recommendations for dates consumption among patients with diabetes and some studies found a deterioration of Type-1 glycemic control.^46^ Physician need to adhere to the daily recommendation of two servings of fruits to avoid the deterioration in blood glucose.^47^

### Strength and limitations:

The strength of this meta-analysis is that it is the first to assess the effects of date’s fruit on dyslipidemia and included a reasonable number of randomized trials. The small number of the included studies, the relatively short duration of the included studies, and the lack of information regarding other confounders limited this study. The current findings should be viewed in the face of the significant heterogeneity observed that may significantly affect the current findings, the small number of the included studies and the lack of standardization of the control diet that closely matches dates. Because of the above, it is difficult to conclude a cause and effect.

We conducted a sub-analysis to address the substantial heterogeniety and found that the source of heterogeniety in cholesterol and triglycerides is due to Al-Albachi S et al.^14^ dates fruit significantly reduced the total cholesterol and triglycerides (odd ratio, -0.59, 95% *CI*, -0.97-0.22, *I*^2^=67, P-value, 0.002, and odd ratio, -0.07, 95% *CI*, -0.09-0.05, *I*^2^=0, P-value< 0.001 respectively) While the source of heterogenity was Ali et al.^16^ in low-density lipoproteins and high-density lipoproteins (odd ratio, -0.12, 95% *CI*, -0.44-0.21, *I*^2^=0, P-value, 0.48, and odd ratio, -0.05, 95% *CI*, -0.14-0.04, *I*^2^=0 P-value, 0.30 respectively).

## CONCLUSION

In this meta-analysis, no significant overall effect of dates fruit was found regarding lipid profile among patients with Type-2 diabetes. Dates fruit could lower total cholesterol, with a non-significant reduction in low-density lipoproteins. No significant effect was evident regarding triglycerides and high-density lipoproteins. Further larger studies with a high selection of controls and dates are needed. Assessing different types of date constituents at different stages of ripening and various cultivation might improve the quality. Larger randomized trials with long duration and combining the effects of dates fruit on lipid profile and glycemic control are highly recommended. Safety concerns regarding heavy metals in some should be addressed.
